# Inequality measures of the generalized gamma distribution with a practicable application tool

**DOI:** 10.1371/journal.pone.0348349

**Published:** 2026-05-19

**Authors:** Emrah Altun, Fulya Gezer, Hana N. Alqifari

**Affiliations:** 1 Department of Statistics, Gazi University, Ankara, Turkey; 2 Department of Econometrics, Ankara Haci Bayram Veli University, Ankara, Turkey; 3 Department of Statistics and Operations Research, College of Science, Qassim University, Buraydah, Saudi Arabia; Pusan National University College of Economics and International Trade, KOREA, REPUBLIC OF

## Abstract

We introduce the inequality measures of the generalized gamma distribution. The Gini coefficient, Theil, Atkinson and Pietra indexes are considered as the inequality measures. The parameters of the generalized gamma distribution are estimated using the minimum distance estimator based on the Kullback-Leibler divergence for the grouped data. The simulation study is constructed to evaluate the performance of the parameter estimation method under the grouped data. The US income data for three different years are analyzed. Also, the GGIneq web-tool is developed to make the application of the inequality measures easy for the practitioners. The GGIneq, accessible via https://smartstat.shinyapps.io/GGIneq/, is a cloud-based system that allows users to obtain inequality measures of the generalized gamma distribution with high-quality graphical results.

## 1 Introduction

Measuring income inequality is important for achieving a more fair distribution of income. There are many measures that are used to measure income inequality. One of the most commonly used measures is the Gini coefficient [1]. The Gini coefficient is calculated by the distance between the Lorenz curve (LC) and the equality line. The Gini coefficient has been used in many fields. [2] evaluated disparities in water supply across India by applying the Gini and Theil indexes to measure inequality. [3] analyzed the global distribution of the nursing workforce using the Gini coefficient to assess inequality across countries. [4] introduced the efficiency Gini coefficient as a novel metric for evaluating technology inequality and identifying diffusion barriers. [5] examined the equity in public transport using the LC, and Gini coefficients. [6] investigated the possible effect of the income inequality and economic growth on the CO_2_ emissions using the panel data modeling.

The other important measure, Pietra, is defined as the maximum vertical difference between the LC and the equality line [7]. [8] obtained the Pietra measures for the exponential, log-normal, beta and Pareto distributions. The excellent work on the Pietra index was carried out by [9] focusing on the McDonalds models such as generalized beta (first and second kinds) and generalized gamma (GG) models. [10] studied on the inequality measures of a new-type Pareto distribution and stated that new-type Pareto distribution is more appropriate than other Pareto distributions when the data has wider range. [11] investigated the income inequality in the Poland using the Gini, Pietra and Theil indexes.

The Theil index, introduced by [12], is also another important measure of income inequality. Since there is a theoretical relationship between the Theil and Atkinson indexes [13], these two measures are often used together. [14] used the Gini and Theil indexes to asses the inequality in water use. [15] analyzed the income inequality in Indonesia using the individual income data based on the Theil decomposition approach. [16] investigated the effect of the home ownership on the income inequality in Turkey using the Atkinson index. [17] emphasised the importance of ensuring equal access to public utilities for the population in order to achieve sustainable development in China, and found inequality in the consumption of public utilities according to city size, based on the results of the Atkinson index.

The aim of this study is to examine income inequality measures for the GG distribution together with theoretical findings. The reason for using the GG distribution is that it is a very comprehensive model that includes log-normal, Weibull, exponential, and gamma distributions as sub-models. Although there are many studies in the literature, the statistical background used to obtain theoretical results has not been sufficiently detailed. For example, [9] examined in detail the derivation of the Pietra index under the GG distribution, but no information is provided regarding the parameter estimation process. [18] examined the Gini coefficient under the GG distribution but did not discuss how to estimate parameters when the number of observations is unknown and only the group proportions are known. In addition, there is no statistical tool that practitioners can use easily to calculate the income inequality measures and obtain LCs. In this study, the GGIneq web application, designed as a cloud-based application in the R Shiny environment, is developed. The household income data typically contain grouped data. Therefore, the GGIneq application estimates the parameters of the GG distribution for grouped data and calculates inequality measures along with the LC.

The remaining sections of the study are summarized as follows. Section 2 discusses inequality measures under the GG distribution. Section 3 presents a parameter estimation method for grouped data and compares the effectiveness of the estimation method for both grouped and raw data through a simulation study. Section 4 demonstrates the use of the theoretical results on the US income data. Section 5 introduces the GGIneq web application. The main findings, information about the future studies, and the limitations of the developed tool are presented in Section 6.

## 2 Generalized gamma distribution and inequality measures

The probability density function (pdf) of the GG distribution is


$$\begin{array}{c} f\left(x;\alpha ,p\right)=\frac{p}{\Gamma \left(\alpha /p\right)}x^{\alpha -1}\exp\left(-x^{p}\right), \end{array}$$ (1)

where α>0 and *p* > 0 are both shape parameters, Γ(·) is the gamma function, and *x* > 0. When the parameter *p* = 1, the GG distribution is equivalent to the gamma distribution with the shape parameter α and fixed scale parameter equal to 1. Also, we have the Weibull distribution for α=p and exponential distribution for α=p=1. For λ>0, if X~GG(α,p,λ), then Y=X/λ, where Y~GG(α,p,1). So, the scale parameter only induces a linear transformation and inequality measures such as the Gini coefficient are scale-invariant. Therefore, fixing λ=1 does not restrict the applicability of the inequality analysis.

**Proposition 1.** *The cumulative distribution function (cdf) of (1) is*


$$\begin{array}{c} F\left(x\right)=\frac{\gamma \left(\alpha /p,x^{p}\right)}{\Gamma \left(\alpha /p\right)}, \end{array}$$ (2)

*where* γ(·) *is the lower incomplete gamma function.*

**Proof 1.** *The cdf of the GG distribution is*


$$\begin{array}{c} F\left(x;\alpha ,p\right)=\int_{0}^{x}\frac{p}{\Gamma \left(\alpha /p\right)}t^{\alpha -1}\exp\left(-t^{p}\right)dt. \end{array}$$ (3)

*Let* *u* = *t*^*p*^, $t=u^{1/p}$ *and* $dt=\frac{1}{p}u^{(1/p)-1}du$*. We have*


$$\begin{array}{cc} F\left(x;\alpha ,p\right) & =\frac{p}{\Gamma \left(\alpha /p\right)}\int_{0}^{x}t^{\alpha -1}\exp\left(-t^{p}\right)dt.\\ & =\frac{p}{\Gamma \left(\alpha /p\right)}\int_{0}^{x^{p}}u^{\left(\alpha -1\right)/p}\exp\left(-u\right)\frac{1}{p}u^{\left(1/p\right)-1}du.\\ & =\frac{1}{\Gamma \left(\alpha /p\right)}\underset{I}{\underbrace{\int_{0}^{x^{p}}u^{\left(\alpha /p\right)-1}\exp\left(-u\right)du.}} \end{array}$$ (4)

*It is obvious that the integration part I is the lower incomplete gamma function,*


$$\begin{array}{cc} F\left(x;\alpha ,p\right) & =\frac{1}{\Gamma \left(\alpha /p\right)}\int_{0}^{x^{p}}u^{\left(\alpha /p\right)-1}\exp\left(-u\right)du.\\ & =\frac{\gamma \left(\alpha /p,x^{p}\right)}{\Gamma \left(\alpha /p\right)}. \end{array}$$ (5)

The mean of the random variable (rv) *X* is


$$\begin{array}{c} E\left(X\right)=\frac{\Gamma \left(\frac{\alpha +1}{p}\right)}{\Gamma \left(\frac{\alpha }{p}\right)}. \end{array}$$ (6)

Let G(x) is the cdf of the gamma distribution. The cdf of the GG distribution can be expressed in terms of the cdf of the gamma distribution, $F\left(x;\alpha ,p\right)=G\left(x^{p},\alpha /p\right)$. So, the quantile function of the GG distribution is


$$\begin{array}{c} Q\left(q;\alpha ,p\right)=Q_{G}{\left(q;\alpha /p\right)}^{\frac{1}{p}} \end{array}$$ (7)

where 0 ≤ *q* ≤ 1 and $Q_{G}\left(q\right)$ is the quantile function of the gamma distribution with shape parameter α/p and scale parameter 1.

**Proposition 2.** *The first moment distribution of the GG is*


$$\begin{array}{c} F_{X_{\left(1\right)}}\left(x\right)=\frac{\gamma \left(\frac{\alpha +1}{p},x^{p}\right)}{\Gamma \left(\frac{\alpha +1}{p}\right)}. \end{array}$$ (8)

**Proof 2.** *The first moment distribution is*


$$\begin{array}{c} F_{X_{\left(1\right)}}\left(x\right)=\frac{p}{\mu \Gamma \left(\alpha /p\right)}\int_{0}^{x}t^{\alpha }\exp\left(-t^{p}\right)dt \end{array}$$ (9)

*Using the same substitutions in the previous proposition, we have*


$$\begin{array}{cc} F_{X_{\left(1\right)}}\left(x\right) & =\frac{p}{\mu \Gamma \left(\alpha /p\right)}\int_{0}^{x}t^{\alpha }\exp\left(-t^{p}\right)dt.\\ & =\frac{p}{\mu \Gamma \left(\alpha /p\right)}\int_{0}^{x^{p}}u^{\alpha /p}\exp\left(-u\right)\frac{1}{p}u^{(1/p)-1}du.\\ & =\frac{1}{\mu \Gamma \left(\alpha /p\right)}\int_{0}^{x^{p}}u^{(\left(\alpha +1\right)/p)-1}\exp\left(-u\right)du.\\ & =\frac{\gamma \left(\frac{\alpha +1}{p},x^{p}\right)}{\mu \Gamma \left(\frac{\alpha }{p}\right)}.\\ & =\frac{\gamma \left(\frac{\alpha +1}{p},x^{p}\right)}{\Gamma \left(\frac{\alpha +1}{p}\right)}. \end{array}$$ (10)

The relationship between the first moment distribution and LC is given by


$$\begin{array}{c} L_{X}\left(u\right)=F_{X_{\left(1\right)}}\left(F^{-1}\left(u\right)\right). \end{array}$$ (11)

Therefore, the LC of the GG distribution is


$$\begin{array}{c} L\left(u\right)=\frac{\gamma \left(\frac{\alpha +1}{p},Q(u,\alpha ,p{)}^{p}\right)}{\Gamma \left(\frac{\alpha +1}{p}\right)}, \end{array}$$ (12)

where Q(u,α,p) is the quantile function of the GG distribution.

**Proposition 3.** *The Gini coefficient of the GG is*


$$\begin{array}{c} G=1-\frac{2\;B_{1/2}\;\left(\frac{\alpha +1}{p},\frac{\alpha }{p}\right)}{B\;\left(\frac{\alpha }{p},\frac{\alpha +1}{p}\right)} \end{array}$$ (13)

*where* $B_{z}(\cdot ,\cdot )$ *denotes the incomplete beta function and* B(·,·) *is the beta function.*

**Proof 3.** *Using the LC, the Gini coefficient can be expressed as follows*


$$\begin{array}{c} G=1-2\int_{0}^{1}L(u)\;du. \end{array}$$ (14)

*As mentioned before, the LC can be written as*


$$\begin{array}{c} L(u)=F_{X_{\left(1\right)}}\;(F^{-1}(u)), \end{array}$$ (15)

*where* $F_{X_{\left(1\right)}}\left(x\right)$ *is the first moment distribution. Let u = F(x), du = f(x) dx, we obtain*


$$\begin{array}{c} G=1-2\int_{0}^{\infty }F_{X_{\left(1\right)}}\left(x\right)f(x)\;dx. \end{array}$$ (16)

*Inserting (1) and (8) in (16), we have*


$$\begin{array}{c} G=1-\frac{2}{\Gamma \left(\frac{\alpha }{p}\right)\Gamma \left(\frac{\alpha +1}{p}\right)}\int_{0}^{\infty }\gamma \;\left(\frac{\alpha +1}{p},x^{p}\right)x^{\alpha -1}e^{-x^{p}}\;dx. \end{array}$$ (17)

*In (17), let* *y* = *x*^*p*^, *so x* = *y*^1/*p*^ *and* $dx=\frac{1}{p}y^{1/p-1}dy$*, and the pdf reduces to the gamma density. Let* a=α/p *and b* = 1/ *p*, *then* $Y=X^{p}\sim Gamma(a,1)$*, and the integral becomes*


$$\begin{array}{c} G=1-\frac{2}{\Gamma (a)\Gamma (a+b)}\int_{0}^{\infty }\gamma (a+b,y)\;y^{a-1}e^{-y}\;dy. \end{array}$$ (18)

*Using the integral representation of the lower incomplete gamma function,*


$$\begin{array}{c} \gamma (a+b,y)=\int_{0}^{y}t^{a+b-1}e^{-t}\;dt, \end{array}$$ (19)

*we have*


$$\begin{array}{c} \int_{0}^{\infty }\gamma (a+b,y)\;y^{a-1}e^{-y}\;dy=\int_{0}^{\infty }\int_{0}^{y}t^{a+b-1}e^{-t}y^{a-1}e^{-y}\;dt\;dy. \end{array}$$ (20)

*Changing the order of integration and applying the transformation y* = *s and t* = *sw where* 0 < *w* < 1, *s* > 0 *and dt dy* = *s dw ds*, *we obtain*


$$\begin{array}{c} \int_{0}^{1}\int_{0}^{\infty }s^{2a+b-1}w^{a+b-1}e^{-s(1+w)}\;ds\;dw. \end{array}$$ (21)

*Using the gamma integration, given below*


$$\begin{array}{c} \int_{0}^{\infty }s^{m-1}e^{-cs}\;ds=\frac{\Gamma (m)}{c^{m}}, \end{array}$$ (22)

*the integration in (21) becomes*


$$\begin{array}{c} \Gamma (2a+b)\int_{0}^{1}\frac{w^{a+b-1}}{(1+w{)}^{2a+b}}\;dw. \end{array}$$ (23)

*Applying* $z=\frac{w}{1+w}$ *transformation in (23), we have*


$$\begin{array}{c} \int_{0}^{1}\frac{w^{a+b-1}}{(1+w{)}^{2a+b}}\;dw=\int_{0}^{1/2}z^{a+b-1}(1-z{)}^{a-1}\;dz=B_{1/2}(a+b,a). \end{array}$$ (24)

*Therefore*,


$$\begin{array}{c} \int_{0}^{\infty }\gamma (a+b,y)\;y^{a-1}e^{-y}\;dy=\Gamma (2a+b)\;B_{1/2}(a+b,a). \end{array}$$ (25)

*Substituting this result into the expression of G, we obtain*


$$\begin{array}{c} G=1-\frac{2\;\Gamma (2a+b)\;B_{1/2}(a+b,a)}{\Gamma (a)\Gamma (a+b)}. \end{array}$$ (26)

*Using the beta function definition*


$$\begin{array}{c} B(a,a+b)=\frac{\Gamma (a)\Gamma (a+b)}{\Gamma (2a+b)}, \end{array}$$ (27)

*we have*


$$\begin{array}{c} G=1-\frac{2\;B_{1/2}(a+b,a)}{B(a,a+b)}. \end{array}$$ (28)

*Changing* a=α/p *and b = 1/p in (28) we obtain the equation in (13).*

The Gini coefficient of the GG distribution was given in the studies of [19] and [18]. As reported in these studies, the Gini coefficient of the GG distribution is


$$\begin{array}{cc} G & =\frac{1}{2^{\frac{2\alpha +1}{p}}B\left(\alpha /p,\left(\alpha +1\right)/p\right)}\\ & \times \left\{\frac{p}{\alpha }{\;}_{2}F_{1}\left(1,\frac{2\alpha +1}{p},1+\frac{\alpha }{p},\frac{1}{2}\right)-\frac{p}{\alpha +1}{\;}_{2}F_{1}\left(1,\frac{2\alpha +1}{p},1+\frac{\alpha +1}{p},\frac{1}{2}\right)\right\}, \end{array}$$ (29)

where _2_*F*_1_ is the Gaussian hypergeometric function (see [20]). The equations (29) and (13) are equivalent to each other. It is easy to verify these equivalence using the relation of the incomplete beta and Gaussian hypergeometric functions, given below


$$\begin{array}{c} B_{x}(a,b)=\frac{x^{a}}{a}\;{\;}_{2}F_{1}(a,1-b;\;a+1;\;x). \end{array}$$ (30)

The hyperg_ 2F1 function in the gsl package of the R can be used to implement the Gaussian hypergeometric function. The other inequality measure, Theil index, is defined as


$$\begin{array}{c} T=E\left(\log\left(\frac{\mu }{X}\right)\right)=-E\left(\log\left(\frac{X}{\mu }\right)\right). \end{array}$$ (31)

**Proposition 4.** *The Theil index of the GG distribution is*


$$\begin{array}{c} T=\log\left(\frac{\Gamma \left(\left(\alpha +1\right)/p\right)}{\Gamma \left(\alpha /p\right)}\right)-\frac{\psi \left(\alpha /p\right)}{p}, \end{array}$$ (32)

*where* ψ(·) *is the digamma function.*

**Proof 4.** *Using the definition of the Theil index, we have*


$$\begin{array}{cc} T & =-E\left(\log\left(\frac{X}{\mu }\right)\right).\\ & =\log\left(\mu \right)-E\left(\log\left(X\right)\right).\\ & =\log\left(\frac{\Gamma \left(\left(\alpha +1\right)/p\right)}{\Gamma \left(\alpha /p\right)}\right)-E\left(\log\left(X\right)\right). \end{array}$$ (33)

*The expected value,* E(log(X))*, is*


$$\begin{array}{c} E[\log(X)]=\int_{0}^{\infty }\log(x)f(x)\;dx=\int_{0}^{\infty }\log(x)\frac{p}{\Gamma (\alpha /p)}x^{\alpha -1}e^{-x^{p}}dx. \end{array}$$ (34)

*To evaluate the integral (34), let* *t* = *x*^*p*^ *and* $dx=\frac{1}{p}t^{1/p-1}dt$*. Then, we have*


$$\begin{array}{cc} \text{E}[\log(X)] & =\int_{0}^{\infty }\log(x)\frac{p}{\Gamma (\alpha /p)}x^{\alpha -1}e^{-x^{p}}dx.\\ & =\int_{0}^{\infty }\log(t^{1/p})\frac{p}{\Gamma (\alpha /p)}t^{(\alpha -1)/p}e^{-t}\frac{1}{p}t^{1/p-1}dt.\\ & =\frac{1}{p\Gamma (\alpha /p)}\int_{0}^{\infty }\log(t)t^{\alpha /p-1}e^{-t}dt. \end{array}$$ (35)

*Now, we use the below identity for the log-moment of the gamma distribution*


$$\begin{array}{c} \int_{0}^{\infty }\log(t)t^{s-1}e^{-t}dt=\Gamma (s)\psi (s),\;s>0, \end{array}$$ (36)

*where* ψ(s) *is the digamma function. Substitute the result of (36) into (35) with* s=α/p, *we have*


$$\begin{array}{c} \text{E}[\log(X)]=\frac{1}{p\Gamma (\alpha /p)}\Gamma (\alpha /p)\psi (\alpha /p)=\frac{1}{p}\psi \left(\frac{\alpha }{p}\right). \end{array}$$ (37)

There is relationship between the Theil and Atkinson indexes. The Atkinson index is defined by


$$\begin{array}{c} A=1-\exp\left(-T\right), \end{array}$$ (38)

where *T* is the Theil index. Inserting (32) into (38), we have


$$\begin{array}{cc} A & =1-\exp\left(\frac{\psi \left(\alpha /p\right)}{p}-\log\left(\frac{\Gamma \left(\left(\alpha +1\right)/p\right)}{\Gamma \left(\alpha /p\right)}\right)\right).\\ & =1+\exp\left(\frac{\psi \left(\alpha /p\right)}{p}\right)\frac{\Gamma \left(\left(\alpha +1\right)/p\right)}{\Gamma \left(\alpha /p\right)}. \end{array}$$ (39)

Pietra index is also popular metric to measure the inequality of the income. Two different approaches are used to calculate the Pietra index. These are based on the LC and incomplete moments. The Pietra index based on the LC is defined as


$$\begin{array}{c} P_{X}=F\left(\mu \right)-L_{X}\left(F\left(\mu \right)\right). \end{array}$$ (40)

The Pietra index based on the first moment distribution is defined as


$$\begin{array}{c} P_{X}=F\left(\mu \right)-F_{X_{\left(1\right)}}\left(\mu \right). \end{array}$$ (41)

So, the computation of the Pietra index under any probability distribution requires F(x), μ and $L_{X}\left(u\right)$ or $F_{X_{\left(1\right)}}\left(x\right)$ [9]. Here, the second approach is used because its computational simplicity. The Pietra index of the GG distribution is


$$\begin{array}{c} P_{X}=\frac{\gamma \left(\alpha /p,{\left(\Gamma \left(\frac{\alpha +1}{p}\right)/\Gamma \left(\frac{\alpha }{p}\right)\right)}^{p}\right)}{\Gamma \left(\alpha /p\right)}-\frac{\gamma \left(\frac{\alpha +1}{p},{\left(\Gamma \left(\frac{\alpha +1}{p}\right)/\Gamma \left(\frac{\alpha }{p}\right)\right)}^{p}\right)}{\Gamma \left(\left(\alpha +1\right)/p\right)}. \end{array}$$ (42)

## 3 Estimation

The data may not be observed as exact values, but rather in grouped form. As mentioned in [21], the maximum likelihood estimators for the grouped data can be obtained by maximizing the following log-likelihood, $\ln\left(N!\right)+\sum_{i=1}^{k}\left(n_{i}\ln P_{i}\left(\alpha ,p\right)-\ln\left(n_{i}!\right)\right)$ where *N* is the total sample size. [22] developed the minimum Cramer-von Mises distance estimator for both complete and grouped data, and derived its asymptotic normality, variance estimation, and robustness properties. [23] compared maximum likelihood, chi-square minimum distance, imputation-based moment estimation, and an ad-hoc method for grouped interval-censored lifetime data and suggested the use of the maximum likelihood and minimum distance estimation methods. [24] proposed an approximate minimum Hellinger distance estimator for grouped continuous data and demonstrated its consistency, asymptotic normality, robustness, and efficiency through theoretical results and simulations.

Suppose the continuous data are divided into *k* mutually exclusive intervals (*a*_*i*_, *b*_*i*_], and instead of raw values, we observe the proportion $\pi_{i}$ of the total sample falling in each interval. The grouped data is defined as


$$\begin{array}{c} \left\{(a_{1},b_{1}],\pi_{1}\right\},\left\{(a_{2},b_{2}],\pi_{2}\right\},\ldots ,\left\{(a_{k},b_{k}],\pi_{k}\right\}, \end{array}$$ (43)

where $\sum_{i=1}^{k}\pi_{i}=1$. Let F(x;α,p) be the cdf of the GG distribution. Then, the probability that an observation falls into the *i*-th interval is


$$\begin{array}{c} P_{i}(\alpha ,p)=F(b_{i};\alpha ,p)-F(a_{i};\alpha ,p). \end{array}$$ (44)

In most instances, only group proportions are observed and likelihood function cannot be constructed. So, the parameters are estimated by minimizing a discrepancy measure between the observed proportions $\pi_{i}$ and the theoretical probabilities $P_{i}(\alpha ,p)$. Here, we use a minimum distance estimator based on the Kullback–Leibler divergence, given in (45).


$$\begin{array}{c} (\hat{\alpha },\hat{p})=\arg\min_{\alpha >0,p>0}\sum_{i=1}^{k}\pi_{i}\log\;\left(\frac{\pi_{i}}{P_{i}(\alpha ,p)}\right). \end{array}$$ (45)

This approach yields consistent parameter estimates and avoids imposing a hypothetical sample size. The Nelder-Mead algorithm, that is available in the optim or mle functions of the R software, is used to minimize the function in (45). The efficiencies of the estimators are discussed via simulation study.

### 3.1 Simulation

A simulation study is performed to demonstrate the efficiencies of the parameter estimation method on both grouped and ungrouped data. The number of simulation replications is set to 1,000. The below parameter vectors are used.

- α=5, *p*=2
- α=2, *p*=1
- α=3, *p*=3

As mentioned in Section 2, the GG distribution reduces to the gamma distribution for *p* = 1 and reduces to the Weibull distribution for α=p. Thus, the second parameter vector shows the simulation results obtained under the gamma distribution, while the third parameter vector shows those obtained under the Weibull distribution.

First, random observations based on the defined sample size are randomly generated from the GG distribution. The generated observations are divided into equal and unequal intervals according to the number of groups, and the number of observations in each group is determined. In order to obtain unequal group intervals, different percentile values of the data are generated randomly. Parameter estimates are obtained for both grouped and ungrouped data. It should be noted that the grouped proportions $\pi_{i}$ are not deterministic quantities. For each simulation replication, the counts $\left(n_{1},\ldots ,n_{k}\right)$ are obtained from the generated sample, and thus the proportions $\pi_{i}=n_{i}/n$ are random variables whose variability depends on the sample size *n*.

The results of the simulation study are interpreted using the estimated mean, bias, mean square error (MSE), relative root mean square error (RRMSE), and mean absolute percentage error (MAPE) values. Four different sample sizes (*n* = 100, 300, 500 and 1000) and two different numbers of groups (*k* = 5 and 10) are used. The results are given in Tables 1–3.

**Table 1:** Simulation results for α=5 and *p* = 2.

k	n	Parameter	Data	Mean	Bias	MSE	RRMSE	MAPE
–	100	α	Ungrouped	5.05708	0.05708	0.24876	0.09975	0.07853
		*p*	Ungrouped	2.00984	0.00984	0.01117	0.05284	0.04202
5		α	Grouped-Equal	5.18416	0.18416	0.35712	0.11952	0.09233
		*p*	Grouped-Equal	2.04224	0.04223	0.01519	0.06163	0.04871
		α	Grouped-Unequal	5.40896	0.40896	0.49896	0.14127	0.11029
		*p*	Grouped-Unequal	2.08788	0.08788	0.02182	0.07387	0.05940
10		α	Grouped-Equal	5.09382	0.09382	0.27296	0.10449	0.08213
		*p*	Grouped-Equal	2.01988	0.01988	0.01189	0.05453	0.04319
		α	Grouped-Unequal	5.40896	0.40896	0.49896	0.14127	0.11029
		*p*	Grouped-Unequal	2.08788	0.08788	0.02182	0.07387	0.05940
–	300	α	Ungrouped	5.03368	0.03368	0.08320	0.05769	0.04664
		*p*	Ungrouped	2.00756	0.00756	0.00361	0.03005	0.02427
5		α	Grouped-Equal	5.07768	0.07768	0.12496	0.07070	0.05701
		*p*	Grouped-Equal	2.01899	0.01899	0.00510	0.03569	0.02863
		α	Grouped-Unequal	5.20833	0.20832	0.15071	0.07764	0.06187
		*p*	Grouped-Unequal	2.04673	0.04672	0.00675	0.04109	0.03277
10		α	Grouped-Equal	5.04280	0.04280	0.09361	0.06119	0.04971
		*p*	Gropued-Equal	2.01040	0.01040	0.00403	0.03173	0.02575
		α	Grouped-Unequal	5.20833	0.20832	0.15071	0.07764	0.06187
		*p*	Grouped-Unequal	2.04673	0.04672	0.00675	0.04109	0.03277
–	500	α	Ungrouped	5.02399	0.02399	0.04934	0.04443	0.03572
		*p*	Ungrouped	2.00433	0.00433	0.00220	0.02345	0.01848
5		α	Grouped-Equal	5.05931	0.05931	0.06663	0.05163	0.04149
		*p*	Grouped-Equal	2.01303	0.01303	0.00290	0.02694	0.02148
		α	Grouped-Unequal	5.14584	0.14584	0.08081	0.05685	0.04505
		*p*	Grouped-Unequal	2.03188	0.03188	0.00372	0.03049	0.02426
10		α	Grouped-Equal	5.02868	0.02868	0.05111	0.04522	0.03614
		*p*	Grouped-Equal	2.00607	0.00607	0.00228	0.02390	0.01889
		α	Grouped-Unequal	5.14584	0.14584	0.08081	0.05685	0.04505
		*p*	Grouped-Unequal	2.03188	0.03188	0.00372	0.03049	0.02426
–	1000	α	Ungrouped	4.99600	−0.00400	0.02356	0.03070	0.02476
		*p*	Ungrouped	1.99850	−0.00150	0.00091	0.01510	0.01231
5		α	Grouped-Equal	5.01229	0.01229	0.03224	0.03591	0.02825
		*p*	Grouped-Equal	2.00266	0.00266	0.00125	0.01766	0.01426
		α	Grouped-Unequal	5.06275	0.06275	0.03581	0.03785	0.03015
		*p*	Grouped-Unequal	2.01432	0.01432	0.00147	0.01917	0.01539
10		α	Grouped-Equal	5.00325	0.00325	0.02510	0.03169	0.02566
		*p*	Grouped-Equal	2.00024	0.00024	0.00100	0.01581	0.01279
		α	Grouped-Unequal	5.06275	0.06275	0.03581	0.03785	0.03015
		*p*	Grouped-Unequal	2.01432	0.01432	0.00147	0.01917	0.01539

**Table 2:** Simulation results for α=2 and *p* = 1.

k	n	Parameter	Data	Mean	Bias	MSE	RRMSE	MAPE
–	100	α	Ungrouped	2.04231	0.04231	0.04626	0.10754	0.08470
		*p*	Ungrouped	1.01117	0.01117	0.00356	0.05970	0.04741
5		α	Gropued-Equal	2.07612	0.07612	0.09571	0.15469	0.12156
		*p*	Gropued-Equal	1.02057	0.02057	0.00598	0.07731	0.06104
		α	Gropud-Unequal	2.18169	0.18169	0.09030	0.15025	0.11689
		*p*	Gropud-Unequal	1.05484	0.05484	0.00745	0.08630	0.06841
10		α	Gropued-Equal	2.05170	0.05170	0.05522	0.11749	0.09222
		*p*	Gropued-Equal	1.01443	0.01443	0.00405	0.06367	0.04968
		α	Gropud-Unequal	2.18169	0.18169	0.09030	0.15025	0.11689
		*p*	Gropud-Unequal	1.05484	0.05484	0.00745	0.08630	0.06841
–	300	α	Ungrouped	2.00759	0.00759	0.01282	0.05661	0.04457
		*p*	Ungrouped	1.00307	0.00307	0.00102	0.03187	0.02549
5		α	Gropued-Equal	2.01618	0.01618	0.03317	0.09106	0.07188
		*p*	Gropued-Equal	1.00619	0.00619	0.00198	0.04452	0.03533
		α	Gropud-Unequal	2.07345	0.07345	0.02189	0.07398	0.05693
		*p*	Gropud-Unequal	1.02465	0.02465	0.00192	0.04382	0.03507
10		α	Gropued-Equal	2.00447	0.00447	0.01658	0.06437	0.05160
		*p*	Gropued-Equal	1.00300	0.00300	0.00120	0.03457	0.02755
		α	Gropud-Unequal	2.07345	0.07345	0.02189	0.07398	0.05693
		*p*	Gropud-Unequal	1.02465	0.02465	0.00192	0.04382	0.03507
–	500	α	Ungrouped	2.00699	0.00699	0.00800	0.04472	0.03588
		*p*	Ungrouped	1.00230	0.00230	0.00065	0.02553	0.02035
5		α	Gropued-Equal	2.00771	0.00771	0.02384	0.07720	0.06094
		*p*	Gropued-Equal	1.00323	0.00323	0.00142	0.03768	0.03009
		α	Gropud-Unequal	2.05346	0.05346	0.01201	0.05480	0.04376
		*p*	Gropud-Unequal	1.01779	0.01779	0.00107	0.03275	0.02617
10		α	Gropued-Equal	2.00606	0.00606	0.01070	0.05172	0.04063
		*p*	Gropued-Equal	1.00234	0.00234	0.00079	0.02817	0.02228
		α	Gropud-Unequal	2.05346	0.05346	0.01201	0.05480	0.04376
		*p*	Gropud-Unequal	1.01779	0.01779	0.00107	0.03275	0.02617
–	1000	α	Ungrouped	2.00390	0.00390	0.00407	0.03190	0.02527
		*p*	Ungrouped	1.00152	0.00152	0.00035	0.01868	0.01481
5		α	Gropued-Equal	2.02199	0.02199	0.01284	0.05666	0.04466
		*p*	Gropued-Equal	1.00537	0.00537	0.00070	0.02648	0.02117
		α	Gropud-Unequal	2.02851	0.02851	0.00583	0.03816	0.03050
		*p*	Gropud-Unequal	1.00954	0.00954	0.00057	0.02386	0.01894
10		α	Gropued-Equal	2.00805	0.00805	0.00566	0.03762	0.02908
		*p*	Gropued-Equal	1.00244	0.00244	0.00042	0.02058	0.01595
		α	Gropud-Unequal	2.02851	0.02851	0.00583	0.03816	0.03050
		*p*	Gropud-Unequal	1.00954	0.00954	0.00057	0.02386	0.01894

**Table 3:** Simulation results for α=3 and *p* = 3.

k	n	Parameter	Data	Mean	Bias	MSE	RRMSE	MAPE
–	100	α	Ungrouped	3.04634	0.04633	0.08424	0.09675	0.07463
		*p*	Ungrouped	3.05658	0.05658	0.07057	0.08855	0.06847
5		α	Gropued-Equal	3.10624	0.10624	0.12102	0.11596	0.08903
		*p*	Gropued-Equal	3.15168	0.15168	0.11434	0.11271	0.08681
		α	Gropud-Unequal	3.21439	0.21439	0.15525	0.13134	0.10103
		*p*	Gropud-Unequal	3.26196	0.26196	0.18094	0.14179	0.10624
10		α	Gropued-Equal	3.06500	0.06499	0.09155	0.10086	0.07824
		*p*	Gropued-Equal	3.09081	0.09080	0.08221	0.09558	0.07294
		α	Gropud-Unequal	3.21439	0.21439	0.15525	0.13134	0.10103
		*p*	Gropud-Unequal	3.26196	0.26196	0.18094	0.14179	0.10624
–	300	α	Ungrouped	3.01793	0.01793	0.02472	0.05241	0.04194
		*p*	Ungrouped	3.01622	0.01622	0.02052	0.04775	0.03759
5		α	Gropued-Equal	3.03867	0.03867	0.03274	0.06031	0.04744
		*p*	Gropued-Equal	3.05386	0.05386	0.02890	0.05667	0.04443
		α	Gropud-Unequal	3.09671	0.09671	0.03922	0.06601	0.05126
		*p*	Gropud-Unequal	3.10868	0.10868	0.03910	0.06592	0.05180
10		α	Gropued-Equal	3.02605	0.02605	0.02646	0.05422	0.04316
		*p*	Gropued-Equal	3.03153	0.03153	0.02284	0.05038	0.03936
		α	Gropud-Unequal	3.09671	0.09671	0.03922	0.06601	0.05126
		*p*	Gropud-Unequal	3.10868	0.10868	0.03910	0.06592	0.05180
–	500	α	Ungrouped	3.01297	0.01297	0.01338	0.03855	0.03080
		*p*	Ungrouped	3.01614	0.01614	0.01202	0.03655	0.02906
5		α	Gropued-Equal	3.02925	0.02925	0.01842	0.04524	0.03576
		*p*	Gropued-Equal	3.04287	0.04287	0.01728	0.04382	0.03425
		α	Gropud-Unequal	3.06682	0.06682	0.02072	0.04799	0.03792
		*p*	Gropud-Unequal	3.08007	0.08007	0.02325	0.05083	0.03889
10		α	Gropued-Equal	3.02062	0.02062	0.01434	0.03992	0.03162
		*p*	Gropued-Equal	3.02606	0.02606	0.01312	0.03819	0.03006
		α	Gropud-Unequal	3.06682	0.06682	0.02072	0.04799	0.03792
		*p*	Gropud-Unequal	3.08007	0.08007	0.02325	0.05083	0.03889
–	1000	α	Ungrouped	3.00550	0.00550	0.00846	0.03065	0.02466
		*p*	Ungrouped	3.00925	0.00925	0.00624	0.02634	0.02077
5		α	Gropued-Equal	3.01115	0.01115	0.00998	0.03329	0.02651
		*p*	Gropued-Equal	3.02141	0.02140	0.00807	0.02995	0.02318
		α	Gropud-Unequal	3.03649	0.03649	0.01178	0.03618	0.02876
		*p*	Gropud-Unequal	3.04831	0.04831	0.01093	0.03486	0.02766
10		α	Gropued-Equal	3.00771	0.00771	0.00895	0.03153	0.02556
		*p*	Gropued-Equal	3.01376	0.01376	0.00660	0.02708	0.02134
		α	Gropud-Unequal	3.03649	0.03649	0.01178	0.03618	0.02876
		*p*	Gropud-Unequal	3.04831	0.04831	0.01093	0.03486	0.02766

The estimated parameters for ungrouped data are consistently the most accurate estimates. As expected, biases, MSE, RRMSE and MAPE decrease monotonically while sample size increases, ensures the consistency of the estimation method. In grouped data, a decrease in estimation accuracy is observed compared to ungrouped data. The situation reflects the information loss caused by the data grouping process. The estimation error arising from the grouping process varies depending on the number of groups. Increasing the number of groups from *k* = 5 to *k* = 10 resulted in a decrease in all evaluation metrics. This finding highlights the importance of the number of groups when estimating the parameters of the GG distribution from grouped data. The choice of grouping scheme also plays an important role. Equal-interval grouping consistently performs better than unequal grouping. Unequal grouping, which is a realistic situation, may assign higher probability values to upper intervals, which can lead to an increase in RRMSE and MAPE values.

Overall, the simulation results demonstrate that while the estimators of the grouped-data are stable for all parameter vectors and sample sizes, their performance is sensitive to the number of groups and structure of data grouping process.

## 4 Application

We use the US family nominal income data for the years 1970, 1975 and 1980. These data sets were reported in [18] and also used by [9]. The same data set is used to calculate the Pietra, Gini, Theil and Atkinson indexes of the GG distribution with LC. The data sets are in grouped format. Therefore, we use the estimation method that is explained in Section 3. Since the gamma and Weibull distributions are the sub-models of the GG distribution, we compare the results of the GG distribution with those of the gamma and Weibull distributions. The estimated parameters of the GG, gamma and Weibull distributions for the data sets are summarized in Table 4.

**Table 4:** Parameter estimates of the GG, gamma and Weibull distributions for three data sets.

Data Sets	Parameters	GG	Gamma	Weibull
1970	α	3.250	9.580	0.334
	*p*	0.673	–	–
1975	α	3.353	12.908	0.296
	*p*	0.629	–	–
1980	α	3.341	18.809	0.248
	*p*	0.573	–	–

The observed proportions are compared with the predicted proportions that are derived from the GG, gamma, and Weibull distributions. The root mean square error (RMSE), mean absolute error (MAE), and MAPE are used as comparison metrics. The results are reported in Tables 5–7. It is evident that the predicted values obtained from the GG distribution are closer to the observed values, and the RMSE, MAE, and MAPE values are smaller than those from the gamma and Weibull distributions.

**Table 5:** Observed and predicted probabilities under GG, gamma and Weibull models for the year 1970.

Intervals	Observed	GG	Gamma	Weibull
<2.5	6.6	4.892	0.051	74.286
2.5-5	12.5	15.113	4.375	7.664
5-7.5	15.2	17.841	22.405	3.969
7.5-10	16.6	16.016	32.703	2.527
10-12.5	15.8	12.802	23.791	1.777
12.5-15	11	9.643	11.231	1.328
15-20	13.1	12.037	5.092	1.864
20-25	4.6	6.008	0.337	1.250
25-35	3	4.325	0.014	1.570
35-50	1.1	1.166	1.030×10^−5^	1.278
50>	0.5	0.157	6.168×10^−11^	2.485
RMSE		0.017	0.072	0.221
MAE		0.015	0.057	0.127
MAPE		0.233	0.741	1.836

**Table 6:** Observed and predicted probabilities under GG, gamma and Weibull models for the year 1975.

Intervals	Observed	GG	Gamma	Weibull
<2.5	3.5	2.333	2.778×10^−4^	73.051
2.5-5	8.5	8.702	0.221	6.950
5-7.5	10.6	12.231	4.302	3.707
7.5-10	10.6	12.867	17.086	2.424
10-12.5	11.4	11.930	27.507	1.748
12.5-15	10.9	10.347	24.928	1.337
15-20	18.8	15.649	22.248	1.935
20-25	11.6	10.073	3.414	1.349
25-35	9.5	10.038	0.293	1.780
35-50	3.2	4.517	0.001	1.559
50>	1.4	1.312	1.129×10^−8^	4.160
RMSE		0.015	0.085	0.226
MAE		0.012	0.073	0.131
MAPE		0.150	0.885	2.571

**Table 7:** Observed and predicted probabilities under GG, gamma and Weibull models for the year 1980.

Intervals	Observed	GG	Gamma	Weibull
<2.5	2.1	0.977	4.146×10^−9^	71.488
2.5-5	4.1	4.089	1.820×10^−4^	5.974
5-7.5	6.2	6.571	0.036	3.283
7.5-10	6.5	7.898	0.785	2.206
10-12.5	7.3	8.339	4.883	1.631
12.5-15	6.9	8.207	13.577	1.276
15-20	14	14.844	44.230	1.903
20-25	13.7	12.083	27.959	1.377
25-35	19.8	16.491	8.424	1.906
35-50	12.8	12.160	0.105	1.789
50>	6.7	8.340	1.452×10^−5^	7.166
RMSE		0.015	0.120	0.227
MAE		0.012	0.093	0.131
MAPE		0.162	0.994	3.620

**Fig 1:**
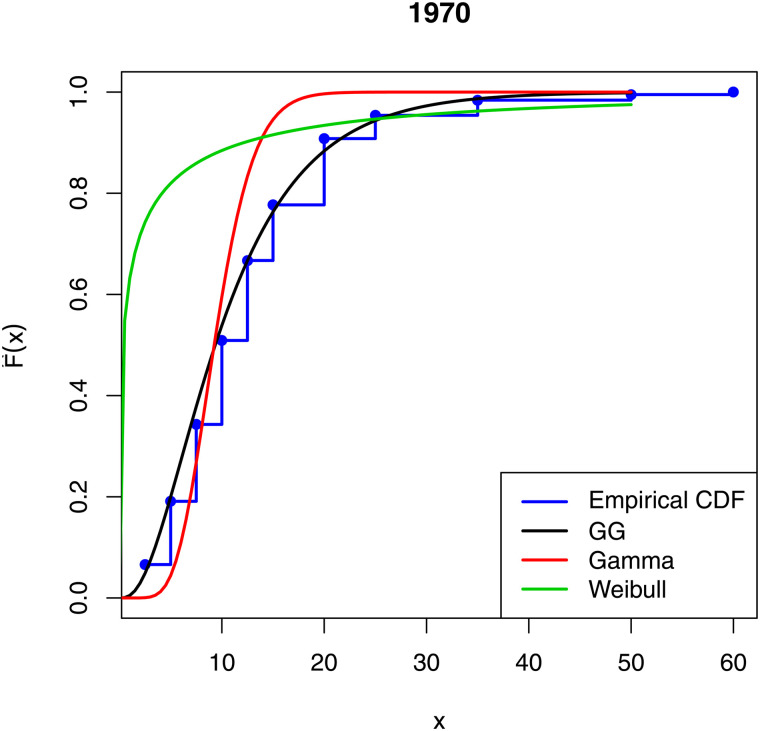
Comparison of empirical distribution functions with estimated cdfs of the fitted models for 1970.

**Fig 2:**
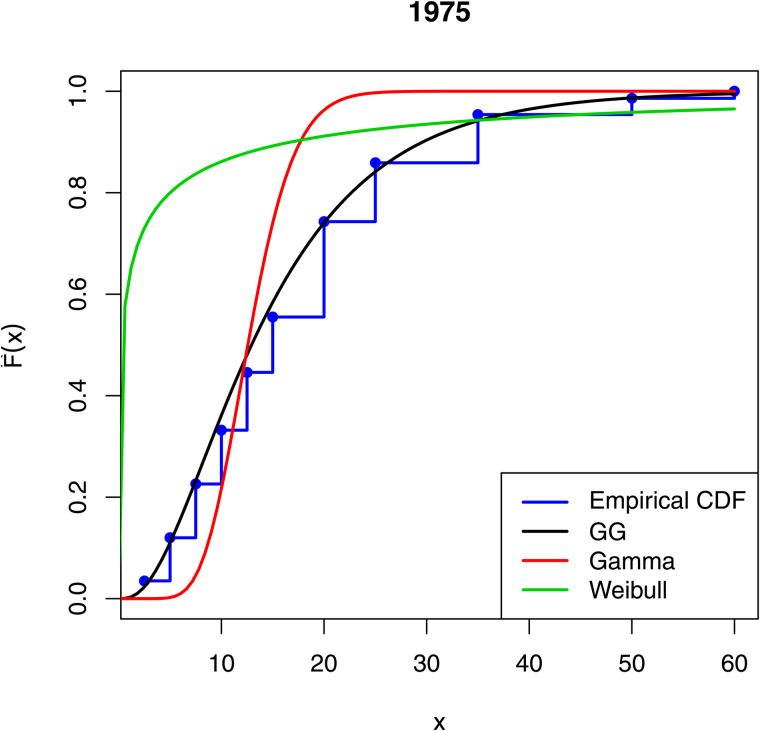
Comparison of empirical distribution functions with estimated cdfs of the fitted models for 1975.

**Fig 3:**
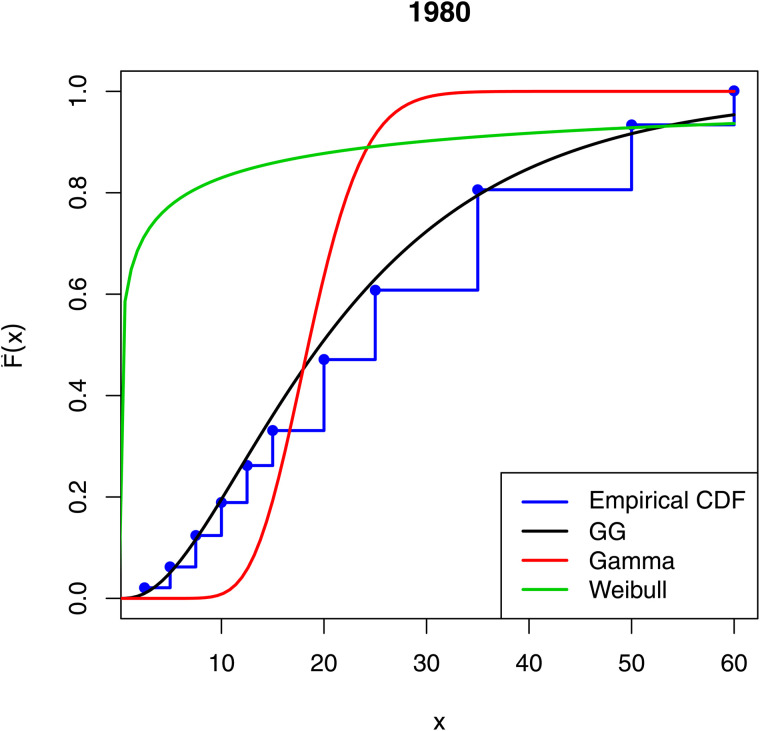
Comparison of empirical distribution functions with estimated cdfs of the fitted models for 1980.

Figs 1–3 compare the empirical distribution function of the data sets with the estimated cdfs of the GG, gamma and Weibull distributions. The excellent fit of the GG distribution to the data is remarkable.

Table 8 shows the estimated values of the Pietra, Gini, Theil and Atkinson indexes obtained under the GG distribution. The results reveal a consistent upward trend in all inequality measures, indicating a significant increase in income disparity over years. Furthermore, when examining the estimated parameters of the GG distribution, the parameter α increases and *p* decreases from 1970 to 1980. Here, the main determining factor is *p* parameter. As the parameter *p* decreases, tail probabilities increase. In other words, as the parameter *p* decreases, income inequality increases.

**Table 8:** Inequality measures estimated by the GG distribution and corresponding bootstrap confidence intervals.

Measures	1970			1975			1980		
	Estimate	Lower	Upper	Estimate	Lower	Upper	Estimate	Lower	Upper
Pietra	0.2567	0.2522	0.2615	0.2609	0.2560	0.2659	0.2725	0.2674	0.2779
Gini	0.3566	0.3506	0.3630	0.3623	0.3556	0.3690	0.3778	0.3709	0.3849
Theil	0.2292	0.2205	0.2385	0.2362	0.2266	0.2463	0.2583	0.2479	0.2694
Atkinson	0.2048	0.1979	0.2122	0.2104	0.2028	0.2183	0.2276	0.2195	0.2362

Differences between years are evaluated using the bootstrap distributions of pairwise differences. A change is considered statistically significant if the corresponding confidence interval does not include zero. The results are given in Table 9. An examination of the results presented in Table 9 reveals that the change in inequality measures between 1970 and 1975 is not statistically significant. However, the difference between the periods 1975–1980 and 1970–1980 is statistically significant.

**Table 9:** Changes in inequality measures over time.

Years	Measures	Lower	Upper	Including zero?
1970-1975	Pietra	−0.01111	0.00265	Yes
	Gini	−0.01488	0.00353	Yes
	Theil	−0.02055	0.00617	Yes
	Atkinson	−0.01626	0.00491	Yes
1975-1980	Pietra	−0.01892	−0.00484	No
	Gini	−0.02525	−0.00646	No
	Theil	−0.03677	−0.00833	No
	Atkinson	−0.02867	−0.00655	No
1970-1980	Pietra	−0.02315	−0.00834	No
	Gini	−0.03089	−0.01116	No
	Theil	−0.04395	−0.01427	No
	Atkinson	−0.03443	−0.01117	No

**Fig 4:**
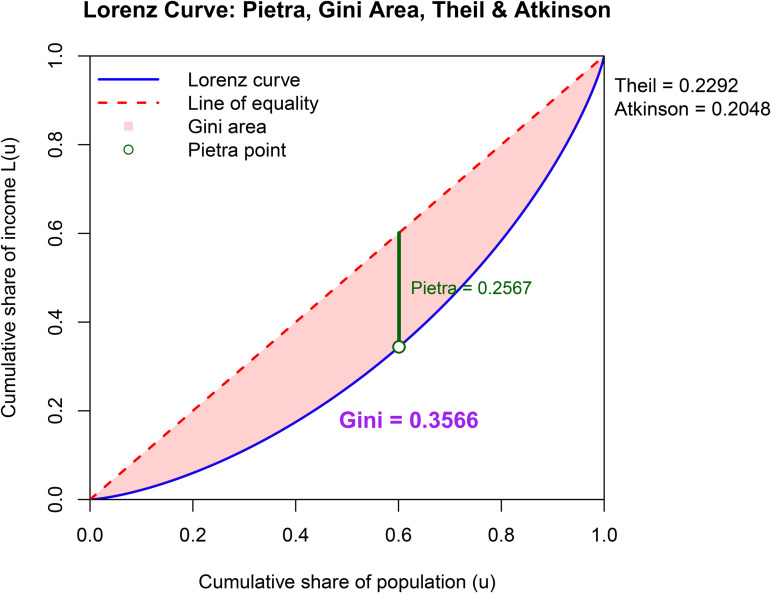
Lorenz curve and inequality measures for 1970.

**Fig 5:**
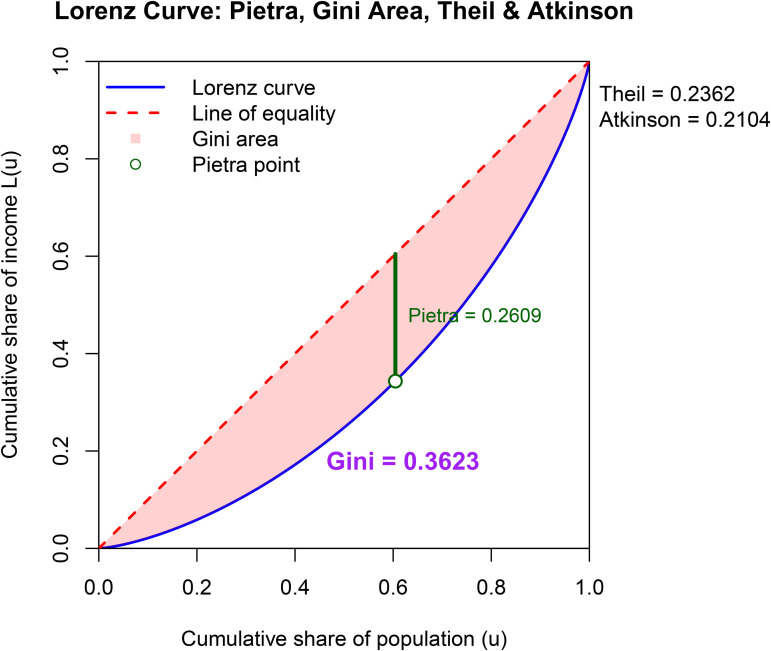
Lorenz curve and inequality measures for 1975.

**Fig 6:**
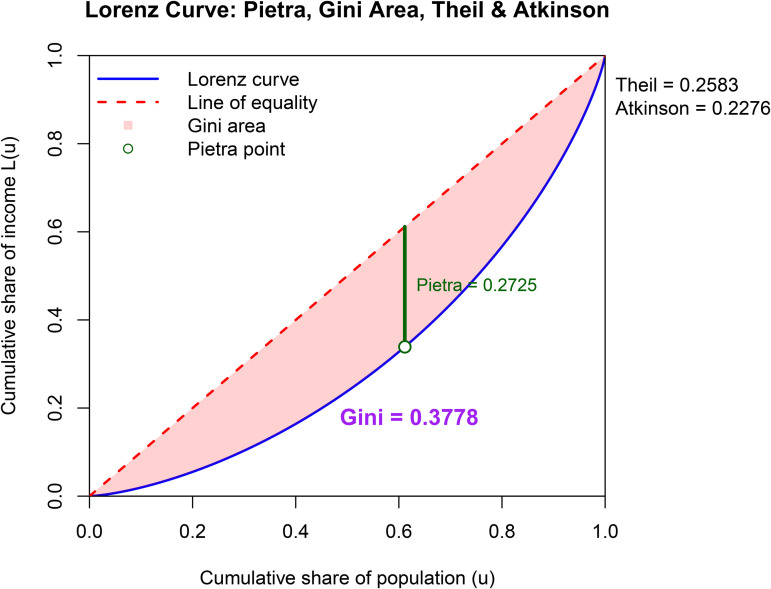
Lorenz curve and inequality measures for 1980.

The graphical presentation of the results is very important in terms of ease of interpretation. In Figs 4–6, four different inequality measures are presented on the LC plots.

## 5 GGIneq web-tool

The GGIneq, available at https://smartstat.shinyapps.io/GGIneq/, is an interactive R Shiny application designed to estimate parameters of the GG distribution based on the grouped data and to compute a set of inequality measures derived from the estimated GG model. The application provides estimated parameters of the GG distribution from grouped observations and reports inequality measures such as Pietra, Gini, Theil, Atkinson with a LC plot that is downloadable in PDF or EPS formats.

**Fig 7:**
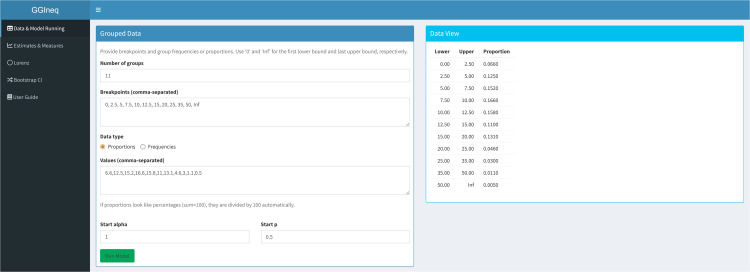
Data entry and parameter initial value setting panel of the GGIneq.

Fig 7 shows the data entry panel of the GGIneq. The application accepts grouped data. The number of groups, breakpoints and proportion or frequency of the each group are provided by the user. The lower bound of the first group starts with 0 and the upper bound of the last group is ended by ∞. Since the application uses Nelder-Mead algorithm, the user should provide the initial values of the parameters. The default values are 1 and 0.5 for α and *p*, respectively.

**Fig 8:**
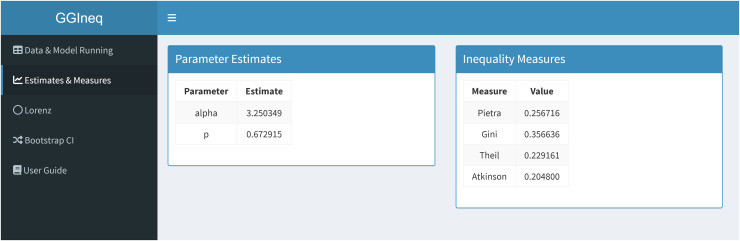
Estimation and inequality measures panel of the GGIneq.

Fig 8 shows the estimated parameters and inequality measures of the GG distribution. The US income data for the year of 1975 is a default data set for the application. Therefore, the results shown in the GGIneq application are the same with the results given in the previous section.

**Fig 9:**
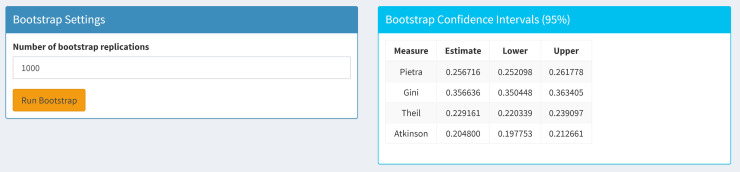
Bootstrap confidence interval panel of the GGIneq.

To assess the sampling variability of the estimated inequality measures, we construct confidence intervals using a parametric bootstrap approach based on the fitted GG distribution. Fig 9 shows the bootstrap panel of the application. The user determines the number of bootstrap replications.

Let $\hat{\alpha }$ and $\hat{p}$ denote the parameter estimates obtained from the grouped data via a minimum distance estimator. The bootstrap samples are generated applying to the following steps:

1. A random sample $\left\{X_{1}^{\left(b\right)},\ldots ,X_{n}^{\left(b\right)}\right\}$ is generated from the GG distribution with parameters $\left(\hat{\alpha },\hat{p}\right)$ using the quantile function of the GG distribution.
2. The simulated observations are grouped using the same class boundaries as the original grouped data, and group proportions are obtained.
3. For each bootstrap sample, b=1,…,B, parameters of the GG distribution, $\left({\hat{\alpha }}^{\left(b\right)},{\hat{p}}^{\left(b\right)}\right)$ are estimated.
4. Using the bootstrap parameter estimates, the inequality measures are computed for each bootstrap sample.

The above procedure yields an empirical bootstrap distribution for each inequality measure. Approximate 95% confidence intervals are obtained using the percentile method, defined by the 2.5th and 97.5th quantiles of the corresponding bootstrap distributions.

**Fig 10:**
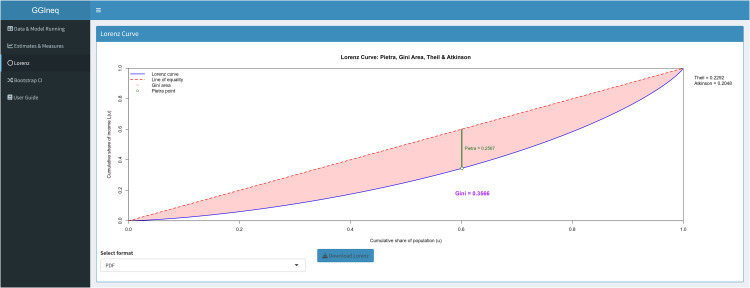
LC plot panel of the GGIneq.

Fig 10 shows the LC plot with inequality measures that can be downloaded in PDF or EPS formats. The LC plot summarizes all the results together without loss of information.

**Fig 11:**
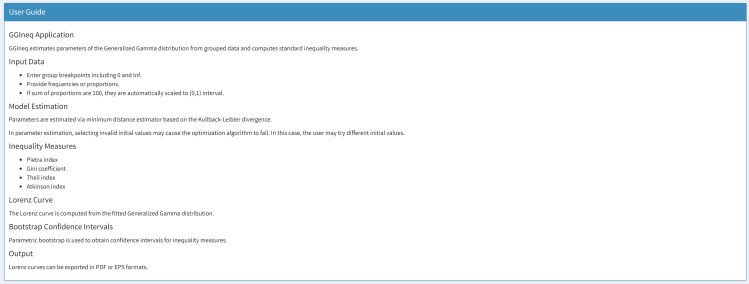
User guide panel of the GGIneq.

The user guide panel, displayed in Fig 11, outlines the key features of the GGIneq application, aiming to prevent user-related issues.

In the development process of the GGIneq, we use several R packages. The shinydashboard [25] package is used to design user interface. The gsl [26] and zifR [27] packages are used for the incomplete gamma and hypergeometric functions. The stats4 package [28] is used for the Nelder-Mead algorithm.

### 5.1 Numerical implementation and considerations

Estimation from grouped data loses within-interval information. For this reason, the estimates may be biased. The evaluation of incomplete gamma and hypergeometric functions may be numerically challenging for extreme parameter values. Therefore, starting values should be chosen carefully. The algorithm may converge to local minimum if starting values are chosen far away from the optimal values. So, users should try multiple starting values when necessary.

The lower bounds of the parameters are set to zero to avoid the negative values. The relative convergence tolerance and maximum iteration are chosen as 10^−8^ and 1,000, respectively. Negative initial values cannot be used in GGIneq, and the system displays a warning message when the user attempts to enter negative initial values for the parameters α and *p*. Additionally, if the optimization algorithm fails to obtain parameter estimates, the system displays another warning message and requests the user to specify different initial values.

To ensure numerical robustness and reproducibility, the application incorporates several protection measures. The input proportions are validated and normalized when necessary. The intervals with zero observed frequency are explicitly handled and excluded from likelihood contributions. To prevent numerical instability in likelihood evaluation, especially under extreme parameter values, small probability thresholds are imposed to avoid undefined logarithmic operations. Furthermore, the optimization routine includes convergence checks. If the algorithm does not converge, the system generates a new initial parameter vector based on the input parameter values and attempts to obtain optimal parameter estimates.

## 6 Conclusion, limitations and future work

In this study, the inequality measures under the GG distribution are examined together with their theoretical foundations. The parameter estimation process of the GG distribution for grouped data is carried out together with a simulation study. An application study is conducted using the US income data. The GGIneq web tool is developed to ensure that the models presented in the study could be used by researchers. It should be noted that the study has several limitations. These are listed below.

✓ The scale parameter of the GG distribution is assumed to be fixed. This assumption is motivated by the fact that standard inequality measures are scale-invariant. However, in empirical applications, researchers may still be interested in estimating and interpreting the scale parameter.
✓ The GGIneq application is designed for grouped data, which leads to information loss relative to individual level data. The estimation accuracy depends on the number and width of the intervals, and the method cannot take the advantage of the individual level data. Moreover, the application does not allow users to explore how inequality measures vary across sub-populations.
✓ The use of the GG distribution imposes a unimodal structure on the income distribution. It may be restrictive in the presence of multimodal income distributions.
✓ The estimation procedure may be sensitive to the upper tail when the data are top-coded which is common in survey-based income data.

To address the limitations of the study, the future research plan can be summarized as follows. The grouping variable feature will be added to the GGIneq application to measure the income inequality within the sub-populations. By adding a data import feature to the GGIneq application, it will be possible to calculate inequality measures based on individual income data. Additionally, users will be able to decide whether the scale parameter of the GG distribution should be fixed or not. So, a more comprehensive tool will be developed for practitioners.
